# Potential implications of mine dusts on human health: A case study of Mukula Mine, Limpopo Province, South Africa

**DOI:** 10.12669/pjms.296.3787

**Published:** 2013

**Authors:** Abuh Momoh, Sphiwe E. Mhlongo, Olukoga Abiodun, Confidence Muzerengi, Matamela Mudanalwo

**Affiliations:** 1 Abuh Momoh, Department of Geology and Mining, University of Jos, Nigeria. Department of Mining and Environmental Geology, University of Venda, South Africa.; 2 Sphiwe E. Mhlongo, Department of Mining and Environmental Geology, University of Venda, South Africa.; 3 Olukoga Abiodun, Department of Public Health, University of Venda, South Africa.; 4 Confidence Muzerengi, Department of Mining and Environmental Geology, University of Venda, South Africa.; 5 Matamela Mudanalwo, Department of Mining and Environmental Geology, University of Venda, South Africa.

**Keywords:** Aggregates, Ambient air, Mine dusts, Suspended particulate matter, Health

## Abstract

***Objective***: The purpose of this study was to estimate the levels of Suspended Particulate Matter (SPM) in ambient air within Mukula mine and the potential risks to mineworkers and inhabitants of the adjoining Mukula community’s health.

***Methods*** ***:*** An SPM was used to measure the levels of particulate matter (PM_10_) in and around the mining site. One-way Analysis of Variance (ANOVA) was used to determine significance level of PM_10 _in ambient air.

***Results***: Suspended particulate matter in the air ranged from 60.25 to 1820.45 µg/m^3^. The lowest value of SPM was more than four times the required World Health Organisation’s allowable level in ambient air, which the mine workers and locals would be inhaling.

***Conclusion***: Continuous inhalation of mine dusts by mine workers and locals could result in pulmonary fibrosis, silicosis and lung cancer. The findings from this study support the need to have in place the necessary control measures that will drastically reduce SPM in the air. Such measure includes wet drilling and blasting, sprinkling of water on the mine roads and planting of vegetation around the mines and neighbouring communities.

## INTRODUCTION

Mine dusts are products of mining activities which are formed when rocks are broken by impact, crushing or grinding. The compositions of these dusts are determined by its source region and closely reflect the composition of soil cover.^1^ These dusts are made of minerals like feldspars, quartz, carbonates, sulphates and phosphates. The dusts are released into different components of the environment (air, water and land) and they could adversely affect the health of mine workers and others living within the vicinity of mining companies.

The total surface area of dust particles are so large that they are more physically, chemically and biologically active than the parent material. Mine dusts are classified based on the size distributions of the particulates and in terms of physiological effects. The physiological effects of mine dusts are further divided into five categories namely; toxic dusts, carcinogenic (cancer causing) dusts; fibrogenic (silicosis causing) dusts; explosive dusts and nuisance dusts.^2^ Particles with the size of 500 - 1000µm get dislodged from the rock surface, but only those with an aerodynamic diameter of less than 7.5µm will get suspended in the atmosphere.^3^ Silicosis is a disease that affects the tissue and space around the air sacs of the lungs due to inhalation of mixed dusts containing silicates.^4^ It occurs in three ways, chronic silicosis which is progressive lung disease characterized by development of scar in response to inhalation of silicates bearing dusts; acute silicosis is the destruction of airways in order for the lungs to become heavy and rigid as air spaces are filled with granular silicate particles and accelerated silicosis which is exposure to silica dusts of almost pure quartz in which the victims show no clinical abnormalities until the condition becomes acute resulting in a decrease of lung function.^5^ According to Jaggard^4^ lead bearing mine dusts with a diameter of < 5µm have a higher potential of being ingested deeper into human lung causing possible tissue damage and toxic effects.

The aim of this present study was to measure the concentration of suspended particulate matter in ambient air and to highlight the potential risks of these dusts to the adjoining Mukula community’s health.

## METHODS

Mukula mine was established by Mukula community members in 2000 to produce aggregates for sales to construction companies. Measurements of dusts in ambient air (PM_10_) were conducted with the aid of a suspended particulate matter (SPM) metre. The readings were taken as composite at four different points so as to arrive at a mean value for specific sampling location. The measurements were carried out at the drilling site at 0m, 20m, 50m, and 100m away, respectively. Data were analysed using a one-way analysis of variance (ANOVA) using a Tukey-Kramer Multiple comparison test.

## RESULTS

Suspended particulate matter in the air ranges from 60.25 – 1820.45 µg/m^3^(Table-I). Highest value of SPM was attained at the drilling point, while lowest value was attained at 100m away from the mine. There was a significant difference (*p* = 0.01112), in the measurements as one moves away from the drilling point towards where the local inhabitants live.

**Table-I:** Dust measurements at drilling points (µg/m^3^).

*Points*	*0 m*	*20m*	*50m*	*100m*
SP 1	1240.01	760.62	215.23	200.45
SP2	630.03	500.41	219.01	200.78
SP 3	630.53	1660.31	230.24	60.25
SP 4	1820.45	890.82	240.36	400.21
Mean	1080.25	953.04	226.21	215.42
SD	571.07	498.07	11.38	139.84

SP = Sampling point, SD = Standard deviation

The least measured SPM was four times greater than the prescribed value for World Health Organization Air Quality Standards. 

## DISCUSSION

Results of the study showed very high concentrations of dusts at the point where whole rock was drilled before crushing them into several sizes of aggregates. World Health Organization considers 55 µg/m^3^as acceptable value and above 90 µg/m^3^ as unacceptable value of dust in ambient air.^6^ Researches have shown that suspended particulate matter is the major causes of asthma, lung cancer, cardiovascular diseases and premature deaths in humans.^1^^,^^6^^,^^7^ The concentrations of SPM at drilling site were more than these prescribed values by WHO. Aggregates that were produced in this mine releases significant amount of PM^10^ into the atmosphere. This finding is consistent with studies in United States of America (USA) and Nigeria.^7^^,^^8^

The implications of these dusts on the health of miners depends on the exposure level, the duration of exposure, the frequency of exposure, the chemical and mineralogical composition of inhaled particle.^3^ Miners were dressed in torn clothes without wearing any personnel protection equipment such as, hard hat, cover all, nose and ear muffs. Researches had shown that when miners are exposed to silica dust over certain period of years. There is always the tendency that these miners would develop silicosis, lung cancer and tuberculosis.^9^

Miners and people living in the Ib valley coal field of India were inhaling fine dust particles of up to 5µm in size and as a result asthma and bronchitis have become a major problem of this community.^10^ In Talcher region of India, high levels of mine dusts from coal fields and other associated minerals were responsible for the occurrence of cancer, tuberculosis, bronchitis and skin diseases.^11^ Mineworkers and inhabitants of Mukula community could be exposed to incidence of these occupational diseases because they live close to this mine that is emitting PM_10_ into the atmosphere in excess of allowable WHO limits.

## CONCLUSION

Mine dusts in ambient air are generally above allowable limits. Prolong exposure to these dusts by miners could lead to respiratory diseases (asthma, silicosis and tuberculosis) and skin disorders. The concentrations of these dusts present great risks to the health of miners and inhabitants of Mukula community around the mine. It is necessary to have control measures such as wet drilling, sprinkling of water on mine roads and planting of vegetation around mine to trap mine dusts. Further studies are required to determine the trace element constituents of mine dusts emission from the Mukula mine.

## References

[1] Portman M (2009). Human respiratory Health effects of inhaled mineral dusts. Term Paper in Environmental Sciences ETH Zurich.

[2] Walli RA (1982). Mine dusts. Mine ventilation and air conditioning.

[3] Smith JL, Lee K (2003). Soil as source of dust and implications for human health. Advan Agro.

[4] Jaggard HC (2012). Mineralogical characteristics of tailings and respirable dust from lead rich mine waste and its control on bioaccessibility. MSc thesis.

[5] Ogola JS, Mitullah W, Omulo MA (2002). Impact of gold mining on the environment and human health: a case study in the Migori gold belt, Kenya. Environ Geochem Hlth.

[6] World Health Organisation (1980). Sixth Report on the World Health Situation.

[7] Engers ED, Smith BF (2002). Environmental Science: A study of interrelationship.

[8] Olusegun O, Aboaba A, Gbadebo TA (2009). Impact of granite quarrying on the health of workers and nearby residents in Abeokuta, Nigeria. Eth J Environ Std and Mgt.

[9] Stephens C, Ahem M (2001). Worker and community health impacts related to mining operations A rapid review of the literature. Min Miner and Sustain Dev.

[10] Sustainable Energy and Economy Network (SEEN) (1996). Coal fired Industrial Colonization of India’s State of Orissa. A report in association with District Action Group.

[11] Mishra PP (2005). Mining and Environmental problems in the Ib valley coal field of Orissa, India. Marker BR.

